# Performance of Pediatrics’ residents as clinical teachers: A student-based assessment

**DOI:** 10.12669/pjms.35.6.830

**Published:** 2019

**Authors:** Muhammad Faheem Afzal, Abrar Ashraf Ali, Asif Hanif

**Affiliations:** 1 Dr. Muhammad Faheem Afzal, FCPS, MHPE. Department of Pediatrics, King Edward Medical University Lahore, Pakistan; 2 Dr. Abrar Ashraf Ali, FCPS, FRCS (Ed), MCPS-HPE, DCPS-HPE, FACS. Department of Surgery, King Edward Medical University Lahore, Pakistan; 3 Asif Hanif, PhD. Department of Biostatistics, University Institute of Public Health, University of Lahore, Lahore, Pakistan

**Keywords:** Peer assisted learning, Near-peer teaching, Resident as teacher, augmented Stanford Faculty Development Program questionnaire

## Abstract

**Objective:**

To assess the clinical teaching skills of Pediatrics’ residents as rated by final year MBBS students by using augmented Stanford Faculty Development Program questionnaire (SFDPQ) in a teaching hospital, Lahore.

**Methods:**

This cross- sectional survey was conducted in the Department of Pediatrics, King Edward Medical University, Lahore in six months in 2016.

Total of 265 students of final year MBBS, attending the teaching sessions organized by residents during their four weeks rotation in Pediatrics were included by non-probability purposive sampling. The augmented SFDPQ was emailed to the study participants after the completion of the clinical rotation, following several encounters with the resident. The data was entered in SPSS 22 for statistical analysis. Scores for each domain (learning climate, control of session, communication of goals, promoting understanding and retention, evaluation, promoting self-directed learning, teacher’s knowledge and teacher’s attitude) were also presented as mean and standard deviation. One-sample Kolmogorov-Smirnov test was applied to observe the normality of data. Where normality of data was observed, independent sample t-test was applied and where normality of data was not observed, Mann-Whitney U test was applied to compare the score between genders. Score of four was considered as cut off score for satisfactory results.

**Results:**

Out of 265 students, 250 responded with response rate of 94.3%. Out of 250 medical students, 105 (42.0%) were male and 145(58.0%) were female. The internal consistency (Cronbach’s alpha) of this score was excellent (0.973). The mean score for all SFDPQ domains was also sub-optimal (2.90±0.611). The mean total score was sub-optimal for learning climate (3.39±0.69), control of session (3.25±0.77), communication of goals (3.26±0.86), promoting understanding and retention (3.26±0.77), evaluation (2.25±0.67), promoting self-directed learning (3.17±0.90), teacher’s knowledge (3.14±0.93) and teacher’s attitude (3.31±0.89), while it was good only for feedback (4.03±0.11). The mean total score for all SFDPQ domains in males and females was 3.05±0.54 and 2.79±0.64 respectively. Although sub-optimal in both the genders, the score was significantly higher in males with p-value 0.001.

**Conclusion:**

We found suboptimal clinical teaching skills of Pediatrics’ residents as rated by final year MBBS medical students.

## INTRODUCTION

Peer assisted learning (PAL) and near-peer learning are rapidly expanding areas of educational research in the field of medical education.^1^,^2^ PAL is defined as “the development of knowledge and skill through active help and support among status equals or matched companions”.^3^,^4^ In cases where tutoring involves experienced students at a more advanced stage of their education (2-5 years ahead of the tutee in learning), the term “near-peer teaching” is more suitable.^5^

The constructs of the interactions in PAL and near peer learning exhibit various teaching behaviors based on the theory of L.S. Vygotsky.^6^ One area that is specifically involved is the “zone of proximal development (ZPD)”, defined as “the distance between the actual developmental level as determined by independent problem solving and the level of potential development as determined through problem solving under adult guidance or in collaboration with more capable peers.” This zone defines the functions that have not yet matured in peers but are in the maturation process.^7^,^8^

Teaching by resident to the undergraduate students is the example of near-peer teachers. Residents are ‘‘consciously competent,’’ as they may deconstruct performance on a clinical task and articulate the detailed steps to facilitate learning by novices and advanced beginners.^3^,^9^ Residents not only find clinical teaching enjoyable but also consider it an important component of their own experience and education.^10^-^12^ Globally, studies have demonstrated that medical students consider residents as one of the important contributory sources of knowledge about patient care.^13^,^14^ Students attribute that approximately, 30% of their overall knowledge is gained from residents’ teaching sessions.^15^,^16^ However, this is important to assess the effectiveness of clinical teaching with respect to its stated objectives and overall impact. This is helpful for meaningful feedback, leading to improving the skills of residents as clinical teachers.^17^,^18^

Little is known about the clinical teaching skills of residents in Pakistan. Studies are needed to identify areas of deficiencies that can be improved. Therefore, this study was planned to assess the clinical teaching skills of Pediatrics’ residents as rated by final year MBBS medical students by using augmented SFDPQ.

## METHODS

This cross-sectional survey was conducted in the department of Pediatrics, King Edward Medical University, Lahore in six months in 2016. Total of 265 students of final year MBBS, attending the teaching sessions organized by residents during their four weeks rotation in Pediatrics were included by non-probability purposive sampling technique. The augmented Stanford Faculty Development Program questionnaire (SFDPQ) was used as student-based assessment tool. This is a validated tool with excellent internal consistency reliability (Cronbach’s alpha = 0.931), and has defined the domains of effective clinical teaching by the residents.^17^,^18^ The augmented SFDPQ was emailed to all the study participants after the fourth week of the clinical rotation, following several encounters with the resident. Although, residents were aware of informal feedback from students to the faculty regarding their teaching skills, but to eliminate a Hawthorne effect, residents were not informed that they are being assessed formally. Those who were relatives of tutor, living with/sharing the residence/hostel or living in the same street were excluded. On the SFDPQ (learning climate, control of session, communication of goals, promoting understanding and retention, evaluation, promoting self-directed learning, teacher’s knowledge and teacher’s attitude), a score of four indicates agreement that the teacher possesses the respective skill, while a score of five indicates excellent demonstration of the corresponding skill. Therefore, interpretation for scores was drawn taking 4 as cut off indicating that a resident possessed good clinical teaching skills for the respective item or domain. The data was entered in SPSS 22 for statistical analysis. Domains and total scores were presented as frequency tables. Scores for each domain were also presented as mean and standard deviation. The internal consistency (Cronbach’s alpha) for augmented SFDPQ was also determined in our circumstances.

### Ethics statement

The study was approved by the Institutional Review Board of King Edward Medical University (137/RC/KEMU on March 10, 2016).

## RESULTS

Out of 265 students, 250 responded with response rate of 94.3%. Out of 250 medical students, 105 (42.0%) were male and 145(58.0%) were female. The internal consistency (Cronbach’s alpha) of this score was excellent (0.973) (Table-I). Taking four as cut off for satisfactory results, mean total score was sub-optimal for learning climate (3.39±0.69), control of session (3.25±0.77), communication of goals (3.26±0.86), promoting understanding and retention (3.26±0.77), evaluation (2.25±0.67), promoting self-directed learning (3.17±0.90), teacher’s knowledge (3.14±0.93) and teacher’s attitude (3.31±0.89). The mean score was good only for feedback (4.03±0.11). The mean score for all SFDPQ domains was also sub-optimal (2.90±0.611). (Table-II)

**Table I:** Effectiveness of Pediatrics’ residents teaching of MBBS students (n=250).

Domains of teaching	No. of items	Cronbach’s Alpha
Learning climate	8	0.823
Control of session	7	0.856
Communication goals	8	0.836
Promoting understanding and retention	8	0.855
Evaluation	7	0.889
Feedback	7	0.915
Promoting self-directed learning	8	0.939
Teacher’s knowledge	5	0.934
Teacher’s attitude	7	0.890
All domains	65	0.973

**Table II:** Total score of different domains of SFDPQ (n=250).

Domain	No of items	Mean+SD	Min-Max	Score < 4
Learning climate	8	3.39+0.69	1.00-4.88	189
Control of session	7	3.25+0.77	1.43-5.00	206
Communication of goals	8	3.21+0.86	1.12-7.12	196
Promoting understanding and retention	8	3.26+0.77	1.00-5.00	196
Evaluation	7	2.25+0.67	0.71-3.57	250
Feedback	7	4.03+1.11	1.29-6.43	114
Promoting self-directed learning	8	3.17+0.90	1.00-5.00	189
Teacher’s knowledge	5	3.14+0.93	1.00-5.00	181
Teacher’s attitude	7	3.31+0.89	1.00-5.00	182
All domains	65	2.90+0.61	1.31-4.19	242

Although compared to female students, male students seemed to be more agreed with learning climate, residents’ skill of promoting understanding and retention, residents’ skill of promoting self-directed learning, teacher’s knowledge and teacher’s attitude, but both the genders seems to be dissatisfied with the mentioned skills. Both male and female students were dissatisfied regarding good control of session, communication of goals, and skill of evaluation by their resident teachers. However, all the students agreed that resident teachers were good in providing feedback (4.25±1.04 in males and 3.86±1.13 in females with p-value 0.005). The mean total score for all domains in males and females was 3.05±0.54 and 2.79±0.64 respectively. Although sub-optimal in both the genders, the score was significantly higher in males with p-value 0.001 (Table-III).

**Table III:** Gender wise performance of teaching skills/domains of pediatric residents (n=250).

Domain	Gender	Mean + SD	Median	IQR	p-value
Learning climate	M =105	3.50+0.70	3.62	1.00	0.016^b^
	F =145	3.31+0.67	3.37	1.06	
	Total =250	3.39+0.69	3.50	0.91	
Control of Session	M =105	3.33+0.73	3.28	0.71	0.150^a^
	F =145	3.19+0.79	3.28	1.00	
	Total =250	3.25+0.77	3.28	0.86	
Communication of goals	M =105	3.37+0.90	3.37	0.88	0.006^b^
	F =145	3.10+0.81	3.12	1.12	
	Total =250	3.21+0.86	3.25	1.03	
Promoting understanding and retention	M =105	3.36+0.68	3.37	1.00	0.092^a^
	F =145	3.19+0.82	3.25	1.00	
	Total =250	3.26+0.77	3.25	1.12	
Evaluation	M =105	2.41+0.60	2.57	0.86	0.002^b^
	F =145	2.14+0.70	2.14	1.14	
	Total =250	2.25+0.67	2.43	1.14	
Feedback	M =105	4.25+1.04	4.43	1.64	0.005^a^
	F =145	3.86+1.13	4.00	1.86	
	Total =250	4.03+1.11	4.14	1.43	
Promoting self-directed learning	M =105	3.39+0.85	3.62	1.00	0.001^b^
	F =145	3.02+0.90	3.25	1.62	
	Total =250	3.17+0.90	3.37	1.50	
Teacher’s Knowledge	M =105	3.32+0.79	3.40	1.40	0.017^b^
	F =145	3.00+1.01	3.20	1.70	
	Total =250	3.14+0.93	3.40	1.60	
Teacher’s attitude	M =105	3.60+0.69	3.71	0.86	<0.0001^b^
	F =145	3.09+0.95	3.14	1.57	
	Total =250	3.31+0.89	3.50	1.29	
All domains	M =105	3.05+0.54	3.08	0.67	0.001^a^
	F =145	2.79+0.64	2.85	1.05	
	Total =250	2.90+0.61	2.93	0.96	

a=Independent sample t-test was applied, b= Mann Whitney U test was applied.

IQR= Inter-quartile range.

## DISCUSSION

Our study is one of the first studies that assessed the clinical teaching skills of Pediatrics’ residents in a Pakistani institution. This study has assessed the clinical teaching skills of Pediatrics’ residents in a Pakistani institution from the perspective of medical students, using a comprehensive formal assessment with Likert scale instrument with established validity. Our survey response rate was excellent (94.3%), which was comparable with the response rate (94%) from the study conducted by Owolabi et al.^17^

In our study, the highest scoring domain was feedback (with mean score of 4.03). In a Dutch study,^19^ in which residents assessed their clinical teachers by using a modified 26-item version of the SFDPQ, communication of goals had the lowest score (mean 5 3.41), and the highest score achieved was 4.07 for ‘‘professional attitude” toward the residents.” Similarly, in study conducted by Owolabi, et al.^17^, the higher scoring domains (with mean score of 3.5) in their assessment were learning climate, teacher’s attitude, and evaluation. Our study has lower scores for all learning domains except feedback. Montacute et al,^20^ identified ten themes among positive and negative reflections regarding residents’ teaching skills, suggesting that the ability to set a safe learning environment is a quality that medical students value in their resident teachers. Professional attitude and feedback were also contesting feature in other authors’ results.^21^-^23^ One possible explanation for this is that medical students’ need for knowledge is being fulfilled by sources other than residents. In turn, residents become more important for establishing a supportive and safe learning climate for medical students, as their near peers, to practice and make mistakes in a nonthreatening environment. Teaching by residents is not part of our curriculum. Residents learn it by their own experience. Therefore, it warrants a clear improvement in our postgraduate curriculum.

The lowest scoring attributes in our study are crucial learning goals, teacher’s ability to present well-organized material, explain relationships in the material, answer learners’ questions clearly, emphasize on specific learning points, internalization of presented learning material, and promote the experiential learning. Our observed performance gaps suggest specific pedagogic learning needs for residents, which are similar to the needs identified in a study of residents in Mexico.^24^ So residents, when assigned a teaching job, should not be sent for “spare time teaching”. They need to be trained for “setting goals” and separate time must be given to them for the preparation.

Although except feedback, there were many differences of rating between males and females, but male students have better rating than female students in every domain. This needs an independent observation by a third commentator about whose (male or female) rating is closer to actual according to the medical education standard. This also needs to be explored that is female dissatisfaction only with resident’s teaching or the same pattern is observed when they are being taught by experienced faculty?

### Limitations of the study

It was conducted in a single site, reducing the generalizability of our findings. Another limitation was partial adherence to Hawthorn effect. However, since the study was conducted every four weeks, and the students of each batch evaluated the residents after completion of their four weeks rotation, the Hawthorne effect was not maintained for the subsequent batches. Moreover, although the responses were obtained after completion of each rotation, but for data analysis purpose, it was analyzed collectively. Despite limitations, we believe that these limitations have not affected our results. Cut off of four as satisfactory score as our pre-determined score for competence in clinical teaching may be too high for residents as teachers. Moreover, our survey consisted of predetermined Likert scale questions which may have prevented the discovery of qualities important to medical students.

### Recommendations

Larger multi-institutional studies are required to highlight factors influencing the clinical teaching skills of resident in our local curriculum. From the results of this study, we can recommend the concerned authorities and stakeholders to integrate formal near peer assisted learning programs in the curriculum of postgraduate residency training program to enhance clinical teaching skills of residents. Furthermore, the augmented SFDPQ, with good internal consistency can be recommended for assessment of teaching skills as well as for the programs aimed at instilling clinical knowledge.

## CONCLUSION

We found suboptimal clinical teaching skills in residents, as of Pediatrics’ residents as rated by final year MBBS medical students. The students rated residents satisfactory for the skill of feedback, whereas rating was suboptimal for learning climate, control of session, communication of goals, promoting understanding and retention, evaluation, promoting self-directed learning, teacher’s knowledge and teacher’s attitude. To enhance clinical teaching skills, formal pedagogic programs should be integrated into residency training.

## Authors’ Contribution:

**MFA, AAA** conceived, designed & editing of manuscript.

**MFA** did data collection and manuscript writing.

**AH** did statistical analysis.

**AAA** did review and final approval of manuscript, is responsible for integrity of research.

## References

[1] Rashid MS, Sobowale O, Gore D (2011). A near-peer teaching program designed, developed and delivered exclusively by recent medical graduates for final year medical students sitting the final objective structured clinical examination (OSCE). BMC Med Educ.

[2] Naqi SA (2014). Peer assisted learning as a formal instructional tool. J Coll Physicians Surg Pak.

[3] Alkhail BA (2015). Near-peer-assisted learning (NPAL) in undergraduate medical students and their perception of having medical interns as their near peer teacher. Med Teach.

[4] Ahsin S, Abbas S, Zaidi N, Azad N, Kaleem F (2015). Reciprocal benefit to senior and junior peers:An outcome of a pilot research workshop at medical university. J Pak Med Assoc.

[5] Tayler N, Hall S, Carr N, Stephens J, Border S (2015). Near peer teaching in medical curricula:integrating student teachers in pathology tutorials. Med Educ Online.

[6] Vygotsky L, Gauvain, Cole Interaction between learning and development. Readings on the development of children.

[7] Jackson TA, Evans DJR (2012). Can medical students teach?A near-peer-led teaching program for year 1 students. Adv Physiol Educ.

[8] Rezaee AA, Azizi Z (2012). The role of zone of proximal development in the students'learning of english adverbs. J Lang Teaching Res.

[9] Snell L (2011). The resident-as-teacher:it's more than just about student learning. J Grad Med Educ.

[10] Berger JS (2012). Anesthesiology residents-as-teachers program:A pilot study. J Grad Med Educ.

[11] Hill AG, Srinivasa S, Hawken SJ, Barrow M, Farrell SE, Hattie J (2012). Impact of a resident-as-teacher workshop on teaching behavior of interns and learning outcomes of medical students. J Grad Med Educ.

[12] Pien LC, Taylor CA, Traboulsi E, Nielsen CA (2011). A pilot study of a “resident educator and life-long learner” program: using a faculty train-the-trainer program. J Grad Med Educ.

[13] Fromme HB (2011). Pediatric resident-as-teacher curricula:A national survey of existing programs and future needs. J Grad Med Educ.

[14] Tuck KK, Murchison C, Flores C, Kraakevik J (2014). Survey of residents'attitudes and awareness toward teaching and student feedback. J Grad Med Educ.

[15] Huynh A, Savitski J, Kirven M, Godwin J, Gil KM (2011). Effect of medical students'experiences with residents as teachers on clerkship assessment. J Grad Med Educ.

[16] Melvin L, Kassam Z, Burke A, Wasi P, Neary J (2014). What makes a great resident teacher?A multicenter survey of medical students attending an internal medicine conference. J Grad Med Educ.

[17] Owolabi MO, Afolabi AO, Omigbodun AO (2014). Performance of residents serving as clinical teachers:a student-based assessment. J Grad Med Educ.

[18] Owolabi MO (2014). Development and psychometric characteristics of a new domain of the Stanford faculty development program instrument. J Contin Educ Health Prof.

[19] Lombarts MJ, Bucx MJ, Rupp I, Keijzer PJ, Kokke SI, Schlack W (2007). An instrument for the assessment of the training qualities of clinician educators. Ned Tijdschr Geneeskd.

[20] Montacute T, Teng VC, Yu GC, Schillinger E, Lin S (2016). Qualities of resident teachers valued by medical students. Fam Med.

[21] Kikukawa M, Nabeta H, Ono M, Emura S, Oda Y, Koizumi S (2013). The characteristics of a good clinical teacher as perceived by resident physicians in Japan:a qualitative study. BMC Med Educ.

[22] Kisiel JB, Bundrick JB, Beckman TJ (2010). Resident physicians'perspectives on effective outpatient teaching:a qualitative study. Adv Health Sci Educ Theory Pract.

[23] Stalmeijer RE, Dolmans DH, Wolfhagen IH, Peters WG, van Coppenolle L, Scherpbier AJ (2010). Combined student ratings and self-assessment provide useful feedback for clinical teachers. Adv Health Sci Educ Theory Pract.

[24] Sa´nchez-Mendiola M, Graue-Wiechers EL, Ruiz-Pe´rez LC, Garcı´a-Dura´n R, Durante-Montiel I (2010). The resident-as-teacher educational challenge:a needs assessment survey at the National Autonomous University of Mexico Faculty of Medicine. BMC Med Educ.

